# Supplementary material for: Structural and Functional Characterization of One Unclassified Glutathione S-Transferase in Xenobiotic Adaptation of Leptinotarsa decemlineata
Source: Int J Mol Sci. 2021 Nov 3;22(21):11921. doi: 10.3390/ijms222111921 (PMC8584303; doi:10.3390/ijms222111921)
Supplement: Supplementary file 1 [file ijms-22-11921-s001.zip › ijms-1452151-supplementary.pdf]

Figure. S1

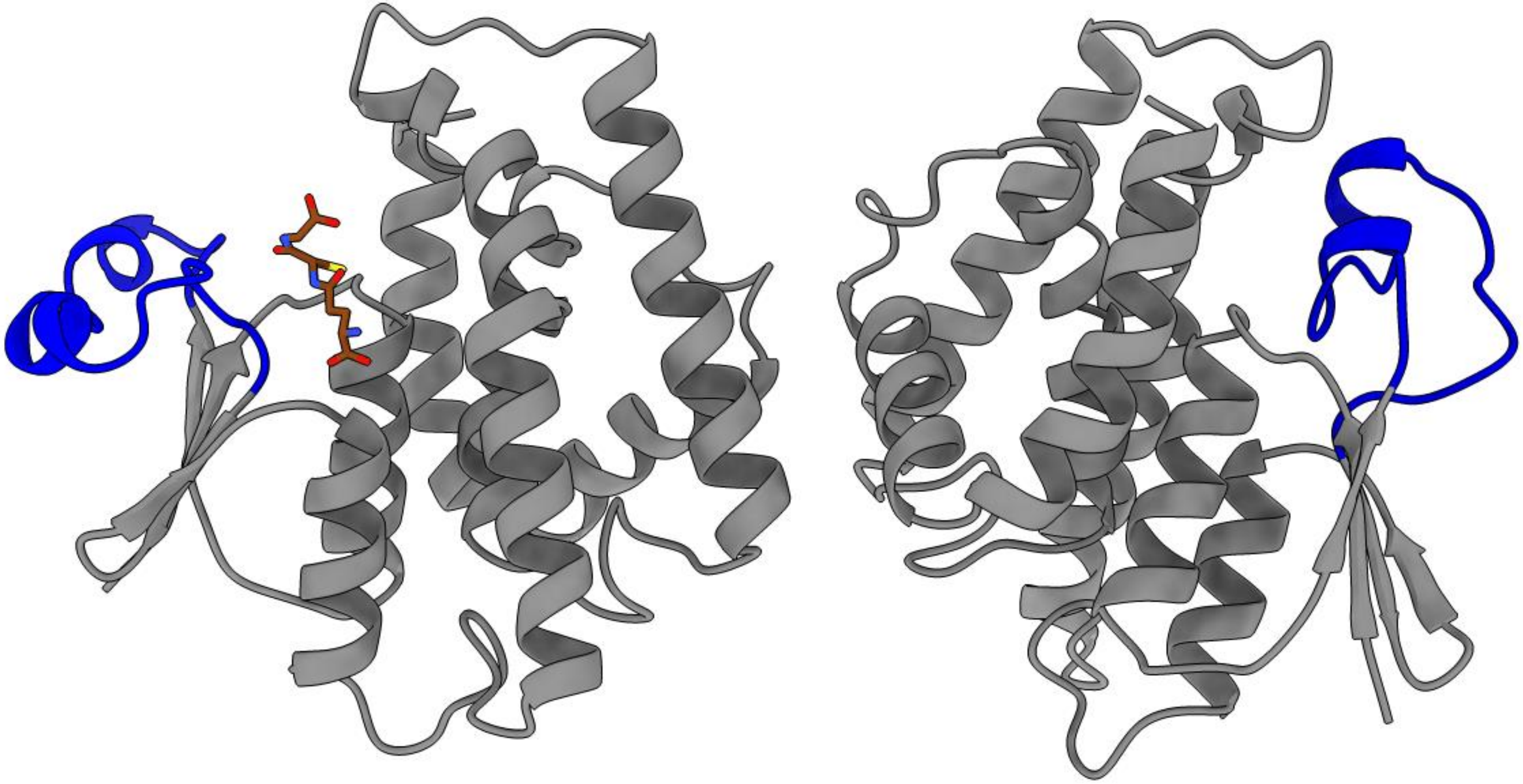

Figure. S2

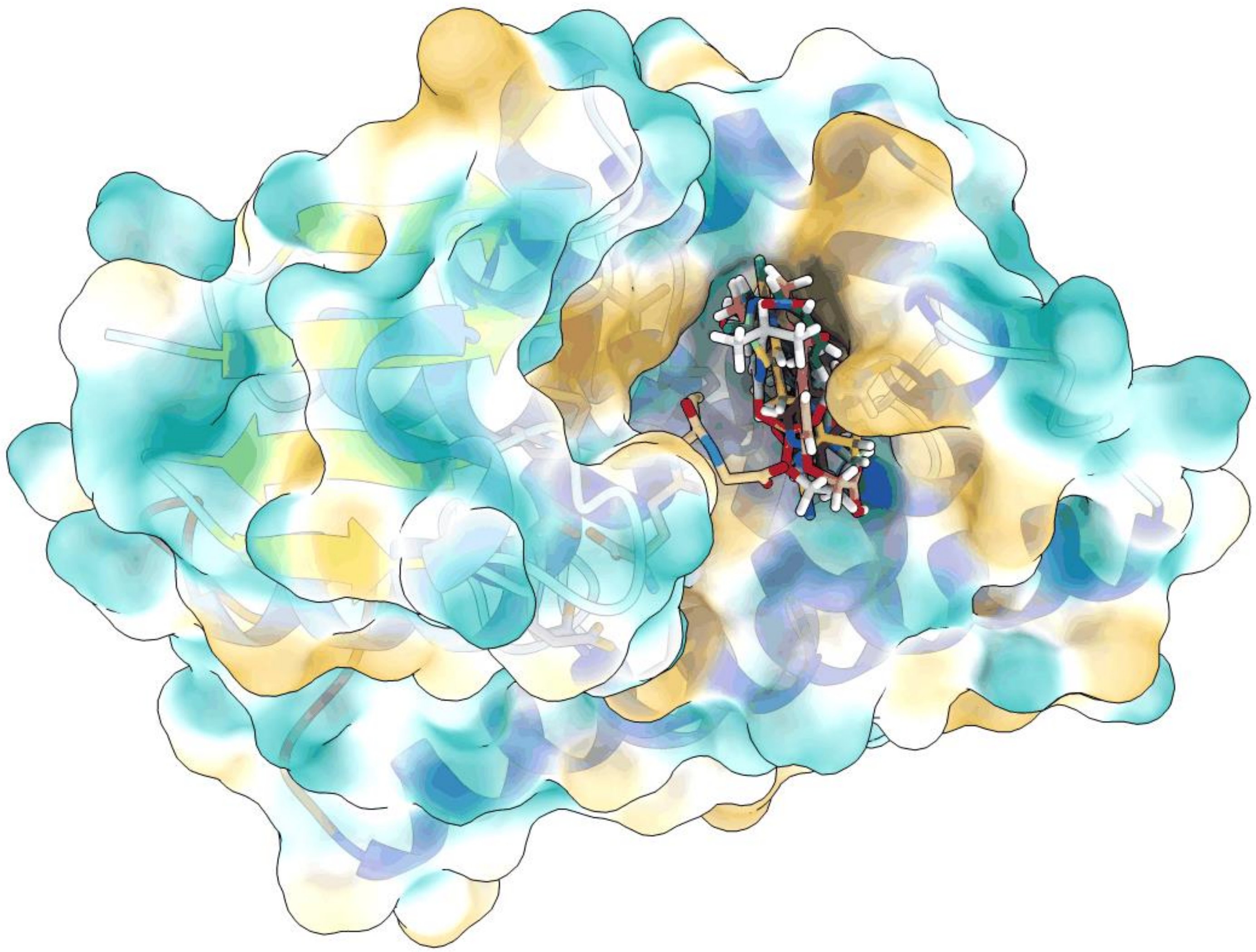

Table S1. Currently available structures of insect GSTs.

Order	Species	PDB ID	GST name	Ligand	Resolution (Å)	Reference	No.
Diptera	*Anopheles dirus*	1JLV	Delta 3	GSH	1.75 Å	Oakely et al., 2001	1
	Anopheles dirus	1JLW	Delta 4	Apo	2.45 Å	Oakely et al., 2001	2
	Anopheles dirus	3F63	Delta 4	GTX	1.80 Å	Wongsantichon et al, 2010	3
	Anopheles dirus	3F6D	Delta 4 F123A	GTX	1.70 Å	Wongsantichon et al, 2010	4
	Anopheles dirus	3G7I	Delta 4	Apo and GSW	2.05 Å	Wongsantichon et al, 2010	5
	Anopheles dirus	3G7J	Delta 4 Y119E	GTX	2.20 Å	Wongsantichon et al, 2010	6
	Anopheles dirus	1R5A	Delta 5	CU	2.50 Å	Udomsinprasert et al., 2005	7
	Anopheles dirus	1V2A	Delta 6	GTS	2.15 Å	Udomsinprasert et al., 2005	8
	Anopheles gambiae	1PN9	Delta 1-6	GTX	2.00 Å	Chen et al., 2003	9
	Anopheles gambiae	2IL3	Epsilon 2	Apo	2.20 Å	Wang et al., 2008	10
	Anopheles gambiae	2IMI	Epsilon 2	GSH	1.40 Å	Wang et al., 2008	11
	Anopheles gambiae	2IMK	Epsilon 2	GTX	1.90 Å	Wang et al., 2008	12
	Anopheles gambiae	4GSN	Epsilon 2	1PE; GOL; GSH	2.30 Å	Mitchell et al., 2014	13
	Drosophila melanogaster	3EIN	Delta 1	GTT	1.13 Å	Low et al., 2010	14
	Drosophila melanogaster	5F0G	Delta 2	K^+; Na^+	1.6 Å	Gonzalez, et al., 2018	15
	Drosophila melanogaster	3F6F	Delta 10	Apo	1.60 Å	Wongsantichon et al, 2010	16
	Drosophila melanogaster	3GH6	Delta 10	GTT	1.65 Å	Wongsantichon et al, 2010	17
	Drosophila melanogaster	1MOU	Sigma 1	GSW	1.75 Å	Singh et al. 2001; Agianian et al., 2003	18
	Drosophila melanogaster	4PNF	Epsilon 6	GSH	2.11 Å	Scian et al., 2015	19
	Drosophila melanogaster	4PNG	Epsilon 7	GSF	1.53 Å	Scian et al., 2015	20
	Drosophila melanogaster	7DB0	Epsilon 14	DC1; DMS	1.66 Å	Koiwai et al., 2021	21
	Scaptomyza nigrita	4I97	Delta 1	GSH	2.15 Å	Gloss et al., 2014	22
	Musca domestica	5ZWP	Delta 1	-	1.40 Å	Sue and Yajima, 2018	23
	Musca domestica	3VWX	Epsilon	GSH	1.80 Å	Nakamura et al., 2013	24
Lepidoptera	*Bombyx mori*	3VK9	Delta	-	2.00 Å	Yamamoto et al., 2012	25
	Bombyx mori	3WD6	Omega	-	2.50 Å	Yamamoto et al., 2013	26
	Bombyx mori	3AY8	Unclassified	-	2.10 Å	Yamamoto et al., 2011	27
	Bombyx mori	5ZFG	Unclassified 2	-	1.70 Å	Yamamoto et al., 2018	28
	Nilaparvata lugens	3WYW	Delta	-	1.70 Å	Yamamoto et al., 2015	29
Coleoptera	*Leptinotarsa decemlineata*	7RKA	Unclassified 1	GSH	1.80 Å	This study	30

Table S2. Primers used in the current study.

Primer name	Primer sequence (5'-3')
LdGSTu1-LIC-Forward	TTTAAGAAGGAGATATAGTTCATGCCAATAACACTCTATTCTG
LdGSTu1-LIC-Reverse	GGATTGGAAGTAGAGGTTCTCTTTCCTCACTGGATGGATG
LdGSTu1-qPCR-Forward	ATGCCAATAACACTCTATTCTG
LdGSTu1-qPCR-Reverse	TTCCAAAATCTACATTCACCAG
EF1α-qPCR-Forward	AAGGTTCCTTCAAGTATGCGTGGG
EF1α-qPCR-Reverse	GCACAATCAGCTTGCGATGTACCA
RPL4-qPCR-Forward	AAAGAAACGAGCATTGCCCTTCCG
RPL4-qPCR-Reverse	TTGTCGCTGACACTGTAGGGTTGA

Table S3. Protein information of GSTs used for phylogenetic analysis.

Insect species	GST name	Accession number	Length (aa)	Reference
Anopheles cracens	AcGSTo1	ACY_95464.1	248	Wongtrakul et al. 2010 JME
Anopheles dirus	AdGSTe1	ABD_43203.1	221	
Anopheles gambiae	AgGSTe1	AAL_59658.1	224	Ortelli et al. 2003. Biochem. J
	AgGSTz1	AAM61889.1	222	Ding et al. 2003. BMC Genomics
Anoplophora glabripennis	AgGST1-1	XP_018564199.1	231	
Apis mellifera	AmGSTd1	NP_001171499.1	217	Elsik et al. 2014. BMC Genomics
	AmGSTt3	XP_624692.2	230	
	AmGSTs4	NP_001136128.1	206	Elsik et al. 2014. BMC Genomics
Bombyx mori	BmGSTe4	NP_001108460.1	217	Yamamoto et al. 2013 Insect Mol Biol
	BmGSTu2	NP_001108462.1	233	Yu et al. 2008 Insect Biochem Mol Biol
	BmGSTt1	NP_001108463.1	229	Yu et al. 2008 Insect Biochem Mol Biol
	BmGSTz1	NP_001037418.1	215	Yu et al. 2008 Insect Biochem Mol Biol
	BmGSTs1	NP_001037077.1	206	Yu et al. 2008 Insect Biochem Mol Biol
Drosophila melanogaster	DmGSTe1	NP_611323.1	224	
	DmGSTd6	NP_524915.1	215	Sun et al. 2006 IMB
	DmGSTo1	NP_648237.1	254	
Drosophila mauritiana	DmGST1-1-X2	XP_033167954	265	
Drosophila mojavensis	DmGST1-X2	XP_015022387.1	231	
Leptinotarsa decemlineata	LdGSTe1	APX61028.1	217	Han et al. 2016 Pesticide Biochem Physiol
	LdGSTd3	APX61027.1	216	Han et al. 2016 Pesticide Biochem Physiol
	LdGSTu1	XP_023027125.1	230	This study

	LdGSTt2	APX61052.1	224	Han et al. 2016 Pesticide Biochem Physiol
	LdGSTs1	APX61045.1	204	Han et al. 2016 Pesticide Biochem Physiol
Locusta migratoria	LmGSTs1	AEB91973.1	204	Qin et al. 2011 Pest Manag Sci
	LmGSTs2	AEB91974.1	204	Qin et al. 2011 Pest Manag Sci
Nasonia vitripennis	NvGSTd1	NP_001165913.1	215	Oakeshott et al. 2010 Insect Mol Biol
	NvGSTt3	NP_001165927.1	221	Oakeshott et al. 2010 Insect Mol Biol
	NvGSTo1	NP_001165912.1	241	Oakeshott et al. 2010 Insect Mol Biol
	NvGSTz1	NP_001165931.1	217	Oakeshott et al. 2010 Insect Mol Biol
Spodoptera litura	SlGSTz2	AIH07599.1	212	
Sitophilus oryzae	SoGST1-X2	XP_030760484.1	239	
